# Fatal complications after an interrupted gastric bypass operation in a patient with non-alcoholic fatty liver disease and massive obesity: a case report

**DOI:** 10.1093/jscr/rjab247

**Published:** 2021-06-23

**Authors:** Pirjo Käkelä, Tuomo Rantanen, Hannu Paajanen, Kirsi A Virtanen

**Affiliations:** Department of Bariatric Surgery, University of Eastern Finland and Kuopio University Hospital, Kuopio, Finland; Department of Surgery, University of Eastern Finland and Kuopio University Hospital, Kuopio, Finland; Department of Surgery, University of Eastern Finland and Mikkeli Central Hospital, Mikkeli, Finland; Department of Public Health and Clinical Nutrition, University of Eastern Finland, Kuopio, Finland; Department of Endocrinology and Clinical Nutrition, Kuopio University Hospital, Kuopio, Finland

**Keywords:** comorbidity, laparoscopy, gastric bypass, liver cirrhosis, NAFLD, NASH

## Abstract

Obesity is closely linked to non-alcoholic fatty liver disease and non-alcoholic steatohepatitis (NASH), the latter now being the most common cause of cirrhosis in Western countries. Only a few cases have been described, such as the unexpected death after interrupted obesity surgery in a patient due to inaccurate preoperative imaging assessment. We describe a 53-year-old male patient with multiple comorbidities partly related to his obesity. A laparoscopic Roux-en-Y gastric bypass (LRYGB) was attempted. During anaesthesia, the patient had a cardiac arrhythmia and a short asystole. Intra-operative findings indicated a giant spleen and, unexpectedly, a cirrhotic liver. The LRYGB operation was interrupted. After 19 months, the patient died due to his severe comorbidities. Preoperative imaging missed the diagnosis of liver cirrhosis and related NASH. Since a challenging liver failure diagnosis cannot only rely on current imaging, we suggest that a liver biopsy is performed prior to LRYGB if preoperative imaging indicates cirrhotic liver.

## INTRODUCTION

Obesity is a major risk factor for non-alcoholic fatty liver disease (NAFLD) [1], although NAFLD can also be present in lean individuals [2]. In severely obese patients, the prevalence of NAFLD is estimated to be 85–98% based on a liver biopsy [1]. NAFLD with obesity increases the risk of pulmonary embolism and various forms of cancer [3], cardiovascular atherosclerosis and atrial fibrillation [4].

Approximately, 10–20% of patients with NAFLD also have non-alcoholic steatohepatitis (NASH) [5]. A NASH diagnosis is challenging without histological analysis. Elevated serum alanine aminotransferase (ALT) and serum aspartate aminotransferase levels may indicate signs of NASH [5], although transaminases can be also normal in patients with NASH [6]. NASH causes a 5- to 6-fold increase in liver-related mortality [7]. Bariatric surgery has been shown to lead to the resolution of NASH in nearly 85% of patients after 1 year in addition to significant reductions in the mean levels of ALT, grade of steatosis, hepatic inflammation, fibrosis and mortality [8]. Portal hypertension increases mortality in NASH [9]. Complications of portal hypertension in cirrhotic patients include oesophageal and gastric varices, ascites and hepatorenal syndrome. The development of portal hypertension in patients with cirrhosis is the primary cause of morbidity and mortality [9].

There is a consensus that all cirrhotic patients should undergo an upper endoscopy as a screening tool for cirrhosis [10]. Computed tomography (CT) and ultrasound (US) can detect oesophageal varices, abdominal venous collaterals, liver steatosis and cirrhosis, changes in liver and spleen size, enlarged portal vein diameter and changes in portal vein flow velocity, which have been found to correlate with the existence of clinically significant portal hypertension [11, 12]. Magnetic resonance imaging (MRI)-related spectroscopy has become the non-invasive gold standard for identifying liver steatosis since it is a reliable tool for assessing the entire liver volume [13]. Since NASH and cirrhosis cannot currently be detected by MRI-related spectroscopy, a liver biopsy may sometimes be necessary.

## CASE REPORT

**Figure 1:**
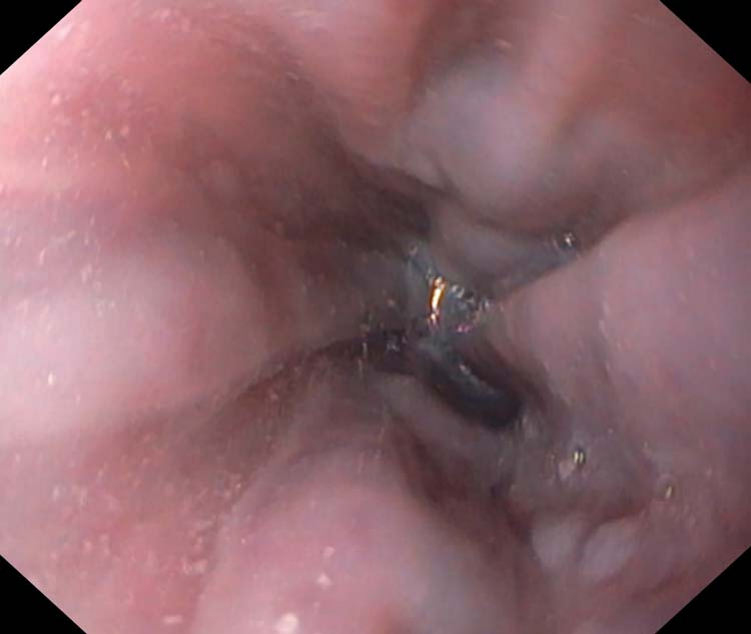
Cesophageal varices 4 months before the LRYGB operation.

**Figure 2:**
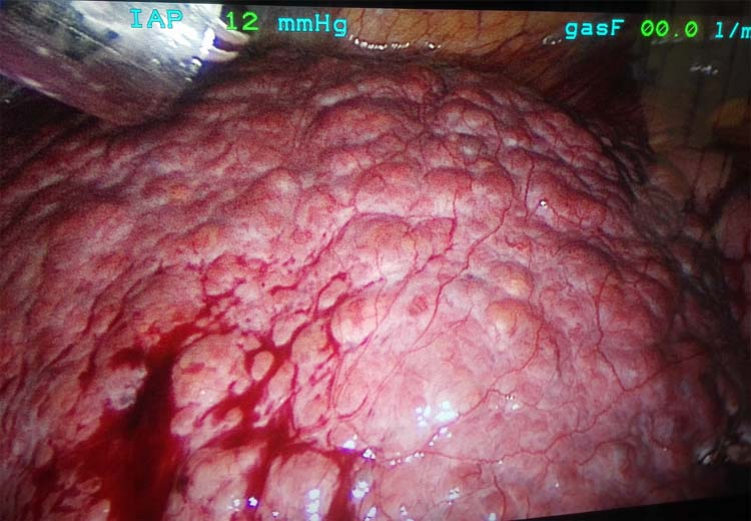
Thick, round-shaped and cirrhotic liver in a patient accepted to LRYGB operation (photographed during the operation by Pirjo Käkelä).

**Figure 3:**
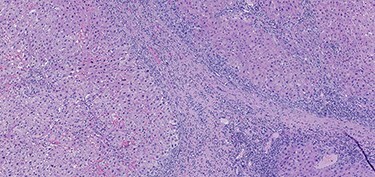
Hematoxylin and eosin stain (H & E) sections of hepatitis (100×).

**Figure 4:**
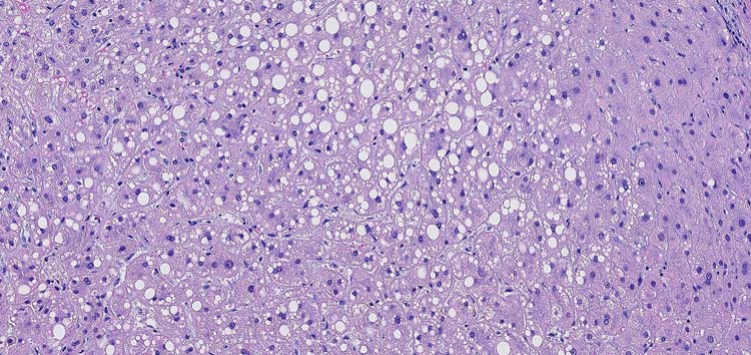
H & E sections of mild steatosis (200×).

**Figure 5:**
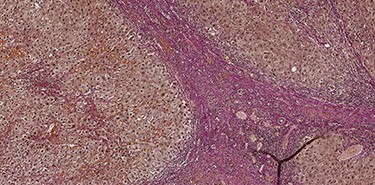
Van Gieson's stain (VG) sections of micronodular cirrhosis (100×).

A 53-year-old male patient was accepted for a laparoscopic Roux-en-Y gastric bypass (LRYGB) operation in November 2016 for morbid obesity (weight: 191 kg, body mass index (BMI): 59 kg/m^2^). The patient had a history of multiple comorbidities, such as hypertension, asthma, rheumatoid arthritis, thrombocytopenia, oesophageal varices, enlarged spleen, pulmonary embolism, deep vein thrombosis in 2007, supraventricular tachycardia, frequent erysipelas, anaemia, hypothyreosis, NAFLD, gastroesophageal reflux disease and gastritis. A routine US of the liver was performed 5 months prior to the operation and no sign of inversion of the portal flow was observed. The anteroposterior axis of the liver was 9 cm, and the longitudinal axis of the left lobe was 2 cm (from the left side of the portal vein). The liver was remarkably fatty, suggesting NAFLD, but not cirrhosis. The longitudinal axis of the spleen was 17 cm. In laboratory values, the serum ALT was 19 IU/l, serum alkaline phosphatase was 75 IU/l, serum thrombocytes were 104 × 10^9^/l, serum bilirubin was 23 μmol/l, serum creatinine was 56 μmol/l, serum albumin (ALB) was 32 g/l and plasma international normalized ratio was 1.2. Gastroscopy was performed routinely 4 months previously. Some oesophageal varices were observed (Fig. 1). The patient fulfilled the criteria for LRYGB. The induction of anaesthesia was unstable because of a cardiac arrhythmia and a short asystole. During laparoscopy, a thick, round-shaped and cirrhotic liver was unexpectedly observed (Fig. 2). The spleen was enlarged and surrounded by abdominal venous collaterals. Intestinal varices as a sign of portal hypertension were observed. The operation was interrupted and a liver biopsy was performed. The patient was discharged the next day. A histopathological study revealed hepatitis, steatosis and micronodular cirrhosis, classified as Child-Pugh A (Figs 3–5). The postoperative period was characterized by fatigue, massive pitting oedema, *Escherichia coli* sepsis, icterus, fever, rheumatic infection, erysipelas, hypoalbuminemia, high portal pressure and severe liver insufficiency with ascites. Hepatitis developed to a more advanced stage Child-Pugh C, decompensated cirrhosis. Unfortunately, 19 months after the interrupted LRYGB operation, the patient died due to his severe comorbidities.

## DISCUSSION

Our patient was accepted to the LRYGB, although there were some risks (oesophageal varices, hypoalbuminemia and thrombocytopenia). He was morbidly obese and had 15 different comorbidities. His liver was remarkably fatty in the preoperative US that was performed 5 months prior to the operation and his portal blood flow was normal. It is possible that the patient already had NASH or cirrhosis, which the US missed. Fibrosis development may occur in NASH, leading to liver cirrhosis in up to 8% of cases [5], and ultimately to death, even after 5 years [5]. A liver biopsy is the gold standard for the diagnosis of NASH, but routine liver biopsies are not taken prior to LRYGB [14]. A CT imaging or US is not always routinely performed. A CT is excellent way of identifying cirrhosis but is not normally used as a screening method because of radiation exposure [15]. The use of MRI-related spectroscopy is limited because it is expensive and is not widely available. It is an excellent tool for identifying liver steatosis, but not NASH and cirrhosis. In our case, routine US was performed 5 months before the LRYGB, and it is possible that the liver failure accelerated between the US and the operation.

However, there were some suspicions of cirrhosis since the patient had oesophageal varices on endoscopy. According to the guidelines, our patient was affected by a Child-Pugh A compensated cirrhosis, and patients with compensated cirrhosis should be sub-staged into those with mild portal hypertension [11]. Although Child-Pugh A cirrhosis does not completely rule out bariatric surgery, we failed to diagnose cirrhosis and elevated portal hypertension. In addition, our patient was encouraged to follow a very low-calorie diet for 5 weeks before the operation with the aim of achieving a 10% weight loss. This could have had a preoperative effect on hypoalbuminemia. In conclusion, a diagnosis of NASH and cirrhosis is sometimes challenging, as indicated by our case report. Furthermore, preoperative diagnosis of liver failure cannot rely only on imaging. We suggest that a liver biopsy is performed prior to LRYGB if preoperative imaging indicates cirrhotic liver.
